# Assessing the Accuracy of International Classification of Diseases (ICD) Coding for Delirium

**DOI:** 10.1177/07334648211067526

**Published:** 2022-02-17

**Authors:** Victoria L Chuen, Adrian C.H. Chan, Jin Ma, Shabbir M.H. Alibhai, Vicky Chau

**Affiliations:** 1 Faculty of Medicine, University of Toronto, Toronto, ON, Canada; 2 Faculty of Medicine, 3710 McMaster University, Hamilton, ON, Canada; 3 Faculty of Medicine, 7235 University of Saskatchewan, Saskatoon, SK, Canada; 4 Biostatistics Research Unit, 7989 University Health Network, Toronto, ON, Canada; 5 Division of General Internal Medicine and Geriatrics, Department of Medicine, 7989 University Health Network, Toronto, ON, Canada; 6 Division of General Internal Medicine and Geriatrics, Department of Medicine, Sinai Health System, Toronto, ON, Canada

**Keywords:** delirium, aged, medical documentation, ICD-10, medical record, hospital discharge

## Abstract

Objective: We assessed the accuracy of the ICD-10 code for delirium (F05) and its relationship with delirium discharge summary documentation. Methods: We performed a retrospective chart review at three academic hospitals. The Chart-based Delirium Identification Instrument (CHART-DEL) was used to identify 108 hospitalized patients aged ≥65 years with delirium, and 758 patients without delirium as controls. We assessed the proportion of patients who received the F05 code and calculated the sensitivity and specificity. We compared the rates of F05 code received between patients with and without “delirium” documented in the discharge summary. Results: Among delirious patients, 46.3% received a F05 code, which has a sensitivity of 46.3% and specificity of 99.6% for delirium. Of charts with “delirium” in the discharge summary (*n* = 67), 67.2% were appropriately coded. Conclusions: Current ICD-10 data inadequately capture delirium. Delirium documentation in the discharge summary is associated with improved delirium coding.

## Introduction

Delirium is an acute disturbance in attention, awareness, and cognition, (American Psychiatric Association, 2013) affecting 29–64% of older adults admitted to medical and geriatric wards (Inouye et al., 2014). The World Health Organization International Classification of Diseases, Tenth Revision (ICD-10) is used to capture diagnoses from patient hospitalizations. These data generate statistics on healthcare utilization and disease burden for administrative, research and funding purposes. Within delirium research, identifying delirium requires prospective clinical assessments using validated tools such as the Confusion Assessment Method (CAM), or the time-intensive CHART-DEL, (Inouye et al., 2005) validated to identify delirium from the chart, in retrospective studies. Using readily available ICD data is enticing but has questionable appropriateness and validity. Previous studies demonstrate exclusive use of ICD data underestimates delirium (Casey et al., 2019; Katznelson et al., 2010).

To our knowledge, there have not been studies comparing the accuracy of ICD-10 coding for delirium with the CHART-DEL. We aimed to address this gap and identify variables associated with accurate ICD-10 coding for delirium.

## Methods

### Setting and Patients

We performed a multi-center retrospective chart review of patients aged ≥65** **years old, consecutively admitted to one of three academic tertiary acute care hospitals in Toronto, Canada between April 1 and June 30, 2016, under a medical (General Internal Medicine/Clinical Teaching Unit, Hospitalist, Neurology, Cardiology) or surgical (General Surgery, Orthopedic Surgery, Cardiac Surgery, Neurosurgery) service. These were included based on their higher estimated prevalence of delirium and comprised both elective and non-elective admissions (Inouye et al., 2014). Our study was approved by our institutions’ research ethics board prior to commencement with a waiver for individual patient consent.

**Figure 1.**
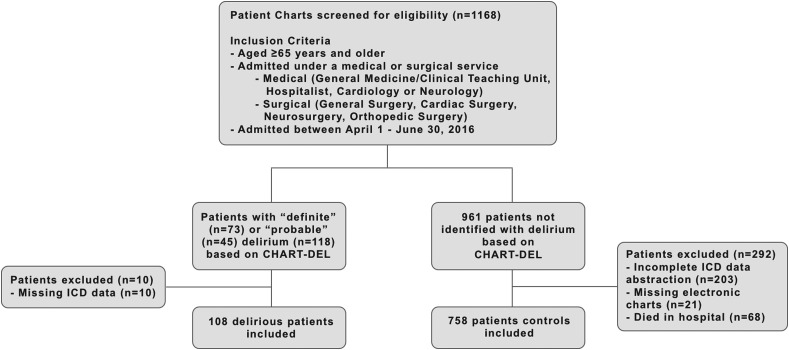
Patient screening, inclusion, and exclusion.

We consecutively screened 1168 charts for delirium using the CHART-DEL (74% sensitivity, 83% specificity) (Inouye et al., 2005). Parallel ratings for 10 charts were completed between an expert rater (i.e., geriatrician) and each researcher. We identified 118 patients with a “Definite” or “Probable” delirium, (Xu et al., 2011) and included 108 patients with ICD-10 data available. Of 961 patients screened negative for delirium, 758 patients were included consecutively as controls, and 203 patients excluded due to incomplete ICD-10 data abstraction. We also excluded patients with missing chart components or who died in hospital, as the latter have abbreviated discharge summaries (Figure 1).

### Data Collection

For delirious patients, we collected demographic data (e.g., age, baseline cognition, functional status, and others). A history of delirium was identified by reviewing the chart for documented delirium dating one year prior. Using hospital administrative ICD datasets, we assessed whether patients received an ICD-10 code for delirium (F05: “Delirium due to known physiological condition,” inclusive of all subheadings within the stem). We defined delirium by the F05 code and its subheadings based on previous studies assessing for delirium through ICD-10 data (Casey et al., 2019; Clegg et al., 2011). We evaluated the proportion of delirious patients who received a code relating to symptoms of delirium or other terminology. As our primary outcome was to evaluate the accuracy of the F05 code, we did not determine *a priori* other codes that could be relevant to delirium. Rather, our ICD-10 dataset included titles of all ICD-10 codes, which allowed us to evaluate their relevance to delirium. In our institutions, ICD-10 codes are assigned by trained administrators, independent of the patient’s clinical team and our research team. Assessment of the ICD-10 codes assigned was completed after CHART-DEL screening to minimize bias during delirium screening.

Of patients identified to have delirium, we assessed each patient’s discharge summary for delirium documentation. This included the use of “delirium” specifically, and other terms, such as “confusion” or “disorientation,” as outlined in Appendix II, CHART-DEL Manual (Xu et al., 2011).

### Statistical Analysis

Using the CHART-DEL as our reference standard to identify delirium, we calculated the sensitivity, specificity, positive predictive value (PPV), and negative predictive value (NPV) of the F05 code. We calculated the proportion of patients with delirium documentation in their discharge summary who received the F05 code and used a chi-squared analysis to assess differences in F05 coding between patients with and without delirium discharge summary documentation.

A univariate and multivariable logistic regression was performed on delirious patients (*n* = 108) to identify factors predictive of receiving a F05 code. Variables were selected a priori, by researcher consensus (two geriatricians with clinical and/or research expertise in delirium). These included patient sex, history of dementia, Charlson Comorbidity Index (CCI), length of stay, delirium symptoms prior to hospitalization, delirium recognition by the attending team, formally diagnosed delirium, delirium type, delirium duration, discharge summary structure, documentation of delirium in the discharge summary, and author of discharge summary. A multivariate logistic regression model using an Akaike Information Criterion stepwise backwards approach determined independent predictors for the F05 code. Model discrimination was calculated with the C-statistic and calibration with the Hosmer and Lemeshow goodness of fit test.

## Results

We included 108 patients with delirium identified from the chart and 758 patients as controls. Patients were on average 79.7 years old and 55.6% were male (Table 1).

**Table 1.** Study population demographic information.

Characteristic	Total (*n* = 108)
Age (years), mean (SD)	79.7 (8.5)
Male sex, *n* (%)	60 (55.6)
English as primary language^a^, *n*(%)	70 (65.4)
Dementia^b^, *n* (%)	29 (26.9)
Baseline functional status^a,c^, *n* (%)	
Independent	28 (29.5)
Impairment in 1–2 domains	43 (45.3)
Dependent in ≥3 domains	24 (25.3)
Pre-admission residence^a,d^, *n* (%)	
Home	82 (76.6)
Nursing home/LTC	13 (12.1)
Retirement home	10 (9.3)
Other	2 (1.9)
Pre-admission social supports^a,e^, *n* (%)	
Alone	22 (27.2)
Alone with external supports	11 (13.6)
Living with family	31 (38.3)
Living with family with external supports	17 (21.0)
History of previous delirium^a^, *n* (%)	31 (33.0)
Charlson comorbidity index^b^, median (IQR)	2.0 (1.0, 4.0)
Number of home medications, mean (SD)	8.8 (4.9)
Length of stay, median days (IQR)	9.0 (5.0, 23.3)

LTC = long term care.

^a^Missing data (*n*). Language (1), Functional status (13), Residence (1), Supports (1), Previous delirium (14). Proportions calculated by excluding missing data from the total sample (*n*=108).

^b^Based on documented past medical history.

^c^Baseline functional status was assessed based on their independence with basic and instrumental activities of daily living (BADLs, IADLs). Independent (no BADL or IADL impairment), impairment (assistance with one to two BADLs or one to two IADLs), dependent (assistance with three or more BADLs).

^d^Other category included complex continuing care, long-term care unit in hospital. Retirement homes are multi-residence living facilities generally for adults ≥65 years, who have their own unit; additional facilities are often provided by the home such as recreational activities or meals. Nursing home and long-term care homes are living facilities for individuals who require assistance for their basic activities of daily living and who have a higher level of medical care required.

^e^Baseline social supports were evaluated for patients residing at home and excluded those living in institutionalized care. External supports included government and privately funded services

^f^Charlson Comorbidity Index was calculated by the research team using chart abstraction data, rather than ICD-10 coding data. Weighting was inclusive of dementia, but not adjusted for age (Charlson et al., 1987).

### Accuracy of the F05 Code for Delirium

Less than half (*n* = 50, 46.3%) of delirious patients identified by the CHART-DEL received the F05 code. Fifty-eight delirious patients did not receive the F05 code (false negatives), and 16 received another related ICD-10 diagnosis or symptom (Table 2). Of patients without delirium, 3 patients received a F05 code, and 755 patients did not (Appendix 1). The F05 code had a sensitivity of 46.3% (95% CI .37–.56), specificity of 99.6% (95% CI .988–.999), PPV of 94.3% (95% CI .84–.99) and a NPV of 92.8% (95% CI .91–.95) for delirium.

**Table 2.** ICD-10 code for delirium and relevant symptoms.

	*n*	Code for delirium (n)				Code for other related diagnosis or symptom (n)								
Presence of delirium		F05.9	F05.1	F05.8	F05.0	F10.4	F22.0	R41.0	R41.88	R41.80	R45.1	R40.0	G93.9	G93.4
Delirium	108	24	3	20	3	1	0	6	3	2	1	1	1	1
No delirium	758	3	0	0	0	0	2	1	1	1	1	1	0	0
Total	866	27	3	20	3	1	2	7	4	3	2	2	1	1
Documentation in Discharge Summary in Delirious Patients (n=108)	n	Code for delirium (n)				Code for other related diagnosis or symptom (n)								
“Delirium”	67	45				4								
Other term	18	1				8								
No documentation	23	4				4								

F05.9 = delirium, unspecified, F05.1 = delirium superimposed on dementia, F05.8 = other delirium, F05.0 = delirium not superimposed on dementia, so described, F10.4 = mental and behavioral disorders due to use of alcohol, withdrawal state with delirium, F22.0 = delusional disorder, R41.0 = disorientation, unspecified, R41.88 = other and unspecified symptoms and signs involving cognitive functions and awareness, R41.80 = transient alteration of awareness, R45.1 = restlessness and agitation, R40.0 = somnolence, G93.9 = disorder of brain, unspecified, G93.4 = encephalopathy, unspecified.

### F05 Coding and Delirium Discharge Summary Documentation

Sixty-seven delirious patients (62.0%) had “delirium” documented in the discharge summary, whereas 85 delirious patients (78.7%) had more broadly, “delirium” or another acceptable term. Charts with “delirium” in the discharge summary received a F05 code significantly more than those without documentation (67.2% vs. 12.2%, *p* < .001). Charts with “delirium” or acceptable term in the discharge summary similarly received significantly more F05 codes compared to charts without documentation (54.1% vs. 17.4%, *p* = .002). Of charts without delirium discharge summary documentation (*n* = 21), 90.5% (*n* = 19) were diagnosed with delirium using the term “delirium”, as documented in the chart somewhere other than the discharge summary.

### Factors Affecting Delirium International Classification of Diseases Coding

The only univariate positive predictor for receiving a F05 code was having “delirium” documented in the discharge summary (OR 9.72, 95% CI (2.95–32.02]) (Table 3). Other variables that negatively predicted F05 coding included hypoactive delirium (OR .25, 95% CI [.09–.74]), delirium being the presenting reason for admission (OR .4, 95% CI [.17–.93]) and increasing CCI (OR .77 per 1-unit increase, 95% CI [.63–.94]).

**Table 3.** Variables affecting the accuracy of ICD-10 Coding of delirium.

Variable^a^	Univariate Odds Ratio (95% CI)	Multivariable Odds Ratio (95% CI)
Male sex	1.03 (0.48–2.21)	
No history of dementia	1.08 (0.46–2.55)	
CCI, per 1-point increase	0.77 (0.63–0.94)	0.68 (0.50–0.93)
LOS, per day	1.01 (0.99–1.03)	1.00 (0.98–1.03)
Delirium as presenting reason for admission	0.4 (0.17–0.93)	
Presence of delirium symptoms prior to admission	0.50 (0.23–1.11)	
Delirium or its symptoms recognized by attending team	1.78 (0.31–10.14)	
Formal diagnosis of delirium made^b^	>100 (CI not calculable)	Not included due to model fit
Delirium type		
Mixed	0.47 (0.18–1.23)	0.27 (0.06–0.45)
Hypoactive	0.25 (0.09–0.74)	0.08 (0.02–0.45)
Hyperactive	Reference level	Reference level
Duration of delirium^c^	1.00 (0.99–1.01)	
Structured discharge summary^d^	0.62 (0.23–1.67)	
“Delirium” documented in discharge summary	9.72 (2.95–32.02)	14.19 (3.11–64.81)
Discharge summary author^e^		
Early trainee	0.55 (0.17–1.80)	
Senior resident	0.55 (0.17–1.72)	
Staff	0.54 (0.14–2.17)	
Other Clinician	Reference level	

CCI = Charlson Comorbidity Index; CI = confidence interval; LOS = length of stay.

^a^Variables were selected based on consensus between researchers.

^b^Diagnosis made by any involved physician, and specifically documented as “delirium.”

^c^Defined as proportion of hospital stay.

^d^Defined as having standardized discharge summary headings (e.g., Chief complaint, History of presenting illness, Medications...etc.).

^e^Early trainee = medical students, Year 1 residents; Senior trainees = Year 2 and higher residents, fellows; Other clinician = physician assistants, nurse practitioners.

Multivariable regression analysis demonstrated having “delirium” documented in the discharge summary was an independent positive predictor for the F05 code (OR 14.19, 95% [CI 3.11–64.81]). Both hypoactive delirium (OR .08, 95% CI .02–.45]) and increasing CCI score (OR .68 per unit, 95% CI [.50–.93]), were independent negative predictors for the F05 code.

## Discussion

Less than half (*n* = 50) of delirious patients identified by the CHART-DEL received the F05 code. The sensitivity of 46.3% is improved from previously reported sensitivities of 3–12%. (Inouye et al., 2005; Katznelson et al., 2010; Pendlebury et al., 2020) We suspect differences are attributed to the reference standard used (CAM vs. CHART-DEL). Inouye et al. found absent delirium documentation increased false negative coding results (Inouye et al., 2005). Our study identified more true positives, related to improved delirium documentation, as selected for by our screening methodology; however, we likely omitted delirious patients with poor chart documentation. Over half (53.7%) of our delirious patients did not receive a F05 code, which illustrates inaccurate coding despite adequate chart documentation. However, 16 delirious patients who did not receive a F05 code received a related ICD-10 diagnosis or symptom (Table 2). Although coders are not permitted to infer diagnoses, 98 of the 108 delirious patients received a diagnosis through the specific term “delirium” as documented in the chart somewhere other than the discharge summary. This significant discrepancy in accurate F05 coding can have implications on our local hospital funding and reported delirium statistics.

The specificity of the F05 code remains high (99.6%), similar to previous (Inouye et al., 2005; Pendlebury et al., 2020). Notably, the sensitivity and specificity reported is for delirium identified by the CHART-DEL as the reference standard. As the CHART-DEL has a sensitivity of 74% for detecting delirium, our reported sensitivity of the F05 code (46.3%) is an overestimate. Therefore, our results support using the F05 code to identify delirium instead of the labor-intensive CHART-DEL. However, the F05 code cannot reliably identify all delirium cases, and therefore, cannot be used to exclude delirium or estimate prevalence or incidence. If used exclusively to identify delirium, researchers must acknowledge the bias toward patients with better delirium documentation.

Although the F05 code has a high PPV (94.3%) and NPV (92.8%) for delirium, both tests are a function of disease prevalence. As delirium prevalence increases, the NPV of the F05 code is expected to decrease. This differs from sensitivity and specificity, which remain unchanged irrespective of prevalence, and therefore are of greater utility. Hence, improving the sensitivity of the F05 code is important and our results support the impact of “delirium” documentation on accurate F05 coding.

A limitation of our study is the ICD-10 outcomes were not used to determine our sample size, as this study was completed in follow-up to another investigation. We also acknowledge our sample inherently captures patients with adequate delirium chart documentation, as this was required for detection by the CHART-DEL. However, not all charts had “delirium” documented in the discharge summary, and when it was, a F05 code was assigned significantly more often. This emphasizes the importance of documenting “delirium” in the discharge summary by the attending team to improve coding accuracy. Another way of improving the accuracy of coding for delirium involves implementing multicomponent interventions as outlined by Pendlebury et al., which increased the sensitivity of the F05 coding for delirium, as identified by the gold standard of prospective clinical assessments using the CAM and DSM criteria, from 12.8% to 60.2% (Pendlebury et al., 2020).

The presence of delirium symptoms at presentation was negatively associated with accurate ICD-10 coding, but not maintained in our multivariable analysis. We emphasize the impact of detailed discharge summaries on accurate coding as our previous study showed delirium symptoms at admission was negatively associated with delirium documentation in the discharge summary (Chuen et al., 2021). Additionally, hypoactive delirium and increasing comorbidity scores were negatively associated with receiving a F05 code in our multivariable analysis. Hypoactive delirium is underrecognized and poorly documented (Voyer et al., 2008). This negatively impacts coding accuracy as coding administrators rely solely on chart information. Our results demonstrate increasing comorbidity by the CCI score was also negatively associated with receiving a F05 code. This conflicts with previous results from Pendlebury et al., where it was associated with improved delirium coding (Pendlebury et al., 2020). This may be attributed to differences in methodology, as our CCI scores were calculated from abstracted chart data rather than ICD-10 codes received, or differences in training or depth of chart review for coding administrators. Another study has also demonstrated more comorbid patients experience greater inaccuracies in coding (Boustani et al., 2010).

The American Health Information Management Association specifies the discharge summary and its listed diagnoses as key components to improving coding accuracy and specificity (Cassidy, 2012). Our results demonstrate delirium documentation in the discharge summary is associated with increased delirium ICD-10 coding accuracy. Efforts are needed to improve delirium documentation in discharge summaries.

Identifying delirium retrospectively is challenging and time consuming. The F05 code for delirium is highly specific for identifying delirium. Unfortunately, the sensitivity is subpar, which limits its utility in estimating delirium incidence or prevalence. In delirious patients, the documentation of “delirium” in the discharge summary was predictive for receiving a F05 code. Therefore, improving the delirium documentation in discharge summaries is one method of improving ICD-10 delirium coding accuracy.

## Supplemental Material

sj-pdf-1-jag-10.1177_07334648211067526 – Supplemental Material for Assessing the Accuracy of International Classification of Diseases (ICD) Coding for Delirium Click here for additional data file. Supplemental Material, sj-pdf-1-jag-10.1177_07334648211067526 for Assessing the Accuracy of International Classification of Diseases (ICD) Coding for Delirium by Victoria L Chuen, Adrian C.H Chan, Jin Ma, Shabbir M.H Alibhai and Vicky Chau in Journal of Applied Gerontology

## References

[bibr1-07334648211067526] American Psychiatric Association (2013). Diagnostic and statistical manual of mental disorders (5th ed.).

[bibr2-07334648211067526] Boustani M. Baker M. S. Campbell N. Munger S. Hui S. L. Castelluccio P. Farber M. Guzman O. Ademuyiwa A. Miller D. Callahan C. (2010). Impact and recognition of cognitive impairment among hospitalized elders. Journal of Hospital Medicine, 5(2), 69–75. 10.1002/jhm.589 20104623 PMC2814975

[bibr3-07334648211067526] Casey P. Cross W. Mart M. W. Baldwin C. Riddell K. Dārziņš P. (2019). Hospital discharge data under‐reports delirium occurrence: Results from a point prevalence survey of delirium in a major Australian health service. Internal Medicine Journal, 49(3), 338–344. 10.1111/imj.14066 30091294

[bibr4-07334648211067526] Cassidy B. (2012). Defining the core clinical documentation set for coding compliance. American Health Information Management Association. https://bok.ahima.org/PdfView?oid=105782

[bibr5-07334648211067526] Charlson M. E. Pompei P. Ales K. L. MacKenzie C. R. (1987). A new method of classifying prognostic comorbidity in longitudinal studies: Development and validation. Journal of Chronic Diseases, 40(5), 373–383. 10.1016/0021-9681(87)90171-8 3558716

[bibr6-07334648211067526] Chuen V. L. Chan A. C. H. Ma J. Alibhai S. M. H. Chau V. (2021). The frequency and quality of delirium documentation in discharge summaries. BMC Geriatrics, 21(1), 307. 10.1186/s12877-021-02245-3 33980170 PMC8117503

[bibr7-07334648211067526] Clegg A. Westby M. Young J. B. (2011). Under-reporting of delirium in the NHS. Age and Ageing, 40(2), 283–286. 10.1093/ageing/afq157 21169280

[bibr8-07334648211067526] Inouye S. K. Leo-Summers L. Zhang Y. Bogardus S. T. Leslie D. L. Agostini J. V. (2005). A chart-based method for identification of delirium: Validation compared with interviewer ratings using the confusion assessment method: chart Identification of Delirium. Journal of the American Geriatrics Society, 53(2), 312–318. 10.1111/j.1532-5415.2005.53120.x 15673358

[bibr9-07334648211067526] Inouye S. K. Westendorp R. G. Saczynski J. S. (2014). Delirium in elderly people. The Lancet, 383(9920), 911–922. 10.1016/S0140-6736(13)60688-1 PMC4120864 23992774

[bibr10-07334648211067526] Katznelson R. Djaiani G. Tait G. Wasowicz M. Sutherland A. M. Styra R. Lee C. Beattie W. S. (2010). Hospital administrative database underestimates delirium rate after cardiac surgery. Canadian Journal of Anesthesia, 57(10), 898–902. 10.1007/s12630-010-9355-8 20645040

[bibr11-07334648211067526] Pendlebury S. T. Lovett N. G. Thomson R. J. Smith S. C. (2020). Impact of a system-wide multicomponent intervention on administrative diagnostic coding for delirium and other cognitive frailty syndromes: Observational prospective study. Clinical Medicine, 20(5), 454–464. 10.7861/clinmed.2019-0470 32934037 PMC7539726

[bibr12-07334648211067526] Voyer P. Cole M. G. McCusker J. St-Jacques S. Laplante J. (2008). Accuracy of nurse documentation of delirium symptoms in medical charts. International Journal of Nursing Practice, 14(2), 165–177. 10.1111/j.1440-172X.2008.00681.x 18315830

[bibr13-07334648211067526] Xu G. Fong T. Yee J. Inouye S. (2011). Delirium identification: A training guide to a chart-based delirium instrument. Hebrew Rehabilitation Center.

