# Supplementary material for: Assessing the Accuracy of International Classification of Diseases (ICD) Coding for Delirium
Source: J Appl Gerontol. 2022 Feb 17;41(5):1485–90. doi: 10.1177/07334648211067526 (PMC9024024; doi:10.1177/07334648211067526)
Supplement: sj-pdf-1-jag-10.1177_07334648211067526 – Supplemental Material for Assessing the Accuracy of International Classification of Diseases (ICD) Coding for Delirium [file sj-pdf-1-jag-10.1177_07334648211067526.pdf]

Appendix 1. Two-by-two table comparing patients identified to have delirium in the chart (using the CHART-DEL) with assignment of the F05 code.

	**Delirium present**	**No delirium**	
**Received F05 Code (Positive test)**	50	3	Total positive tests = 53
**Did not receive F05 Code (Negative test)**	58	755	Total negative tests = 813
	Total with delirium = 108	Total without delirium = 758	Total population = 866
